# Species Traits Predict Assemblage Dynamics at Ephemeral Resource Patches Created by Carrion

**DOI:** 10.1371/journal.pone.0053961

**Published:** 2013-01-11

**Authors:** Philip S. Barton, Saul A. Cunningham, Ben C. T. Macdonald, Sue McIntyre, David B. Lindenmayer, Adrian D. Manning

**Affiliations:** 1 Fenner School of Environment and Society, The Australian National University, Canberra, Australian Capital Territory, Australia; 2 Australian Research Council Centre of Excellence for Environmental Decisions, The Australian National University, Canberra, Australian Capital Territory, Australia; 3 National Environmental Research Program, The Australian National University, Canberra, Australian Capital Territory, Australia; 4 Ecosystem Sciences, Commonwealth Scientific and Industrial Research Organisation, Canberra, Australian Capital Territory, Australia; 5 Land and Water, Commonwealth Scientific and Industrial Research Organisation, Canberra, Australian Capital Territory, Australia; Jyväskylä University, Finland

## Abstract

Carrion is an ephemeral and spatially patchy resource that supports a diverse subset of species linked to nutrient cycling and the decomposition process. A number of studies have separately documented changes in the diversity of plants, arthropods and vertebrates at individual carcasses, but there are few studies that have examined how functional traits of different groups of organisms underpin their responses to carrion patches. We used a carrion addition experiment to compare changes in composition and functional traits of insect and plant assemblages at carcasses compared with control sites. We found that significant changes in insect assemblage evenness and heterogeneity was associated with species’ dispersal traits, and that plant assemblage responses to subsequent soil nitrogen changes was most apparent among graminoids and exotic species. Beetles at carcasses were twice as large as their counterparts at control sites during the first week of carrion decomposition, and also had higher wing loadings. Plants with high specific leaf area responded faster to the carcass addition, and twice as many species recolonised the centre of carcasses in exotic-dominated grassland compared with carcasses in native-dominated grassland. These results provide an example of how traits of opportunist species enable them to exploit patchy and dynamic resources. This increases our understanding of how carcasses can drive biodiversity dynamics, and has implications for the way carrion might be managed in ecosystems, such as appropriate consideration of spatial and temporal continuity in carrion resources to promote heterogeneity in nutrient cycling and species diversity within landscapes.

## Introduction

The transfer of energy and recycling of nutrients via the decomposition of organic matter is a central unifying process that links all organisms to the functioning of ecosystems [1]. However, dead organic matter varies enormously in its spatial and temporal distribution [2], and this determines the magnitude of its contribution to nutrient cycling [3] and the diversity and dynamics of its consumers [4]. Animal carrion is the most nutrient-rich form of dead organic matter [5], and recent reviews have highlighted the overlooked role of carrion in food webs [6], [7], [8], and driving variation in biodiversity and ecological processes in landscapes [5], [9]. Widespread changes to the population dynamics of large vertebrates, through loss of top predators [10] or hunting and harvesting of wild herbivores [11], are affecting the distribution and input of carrion resources in some terrestrial ecosystems [6], [12]. This creates a strong imperative to establish a deeper understanding of the links between carrion, biodiversity and ecosystem functioning.

Ephemeral resource patches, such as rotting logs and dung pads, occur in ecosystems worldwide and are focal points of nutrient cycling and aggregation of invertebrates [13], [14]. Carrion is distinct from many other ephemeral resources due to its faster decomposition rate, and its patchy spatial occurrence [9]. This means that carrion has very different spatial and temporal dynamic to other ephemeral resources, and suggests that carrion may have a disproportionate effect on biodiversity and nutrient cycling in ecosystems.

The decomposition of carrion affects its surroundings through pathways to vertebrate and invertebrate scavengers and detritivores, and their predators, as well as through microbial and fungal decomposers and plants. This generates a temporally dynamic patch with high nutrient concentration and biological activity that contrasts strongly with the nearby environment [5], [15]. To date, studies on the effects of carrion on variation in biodiversity have been restricted to single groups of organisms, such as the detailed examination of successional patterns of arthropods [16], [17], and its localised effects on soil nitrogen or plant growth and composition [15], [18]. This has resulted in a detailed understanding of the specialised role that many insect groups have in carrion decomposition, including the critical role of fly larvae [19], [20], [21], and the complex food webs of associated predators and detritivores [16], [22], [23], [24]. Studies on soil and plant responses have demonstrated that the significant spike in soil nitrogen at a carcass can be detected in nearby plant tissue [15], [25], leading to changes in plant growth and composition [18], [26], and is likely moderated by soil microbial activity [5]. Key studies have also shown the importance of carrion for soil nitrogen and plant dynamics across whole ecosystems [27], [28]. Nevertheless, there are only a handful of comprehensive studies that have integrated multiple groups of organisms into the one study design [3], and there are fewer still that have specifically examined the functional traits of different species that respond to carrion. This leaves much to learn about what traits characterise carrion opportunists, and therefore what traits of species can help us understand the links between ephemeral carrion patches and its effect on variation in biodiversity within landscapes [9].

We present data from an experiment designed to answer a number of questions about how ephemeral carrion resource patches generates variation in ecological communities. We did this by quantifying the responses of different aspects of the carrion food web, including components of the insect community, soil nutrients, and the plant community. We sought to identify the traits associated with insect responses to the patchiness of carrion, and the responses of plants to subsequent changes in soil nutrients. To do this, we first examined changes in assemblage diversity and composition in response to the experimental addition of animal carcasses. Second, we examined whether assemblage changes corresponded with particular combinations of traits, for both insects and plants. We predicted that insect responses would be associated with their dispersal ability, with specialist species having wing morphologies that enable the rapid location of new carrion resources. We also predicted that plant responses would be associated with their growth potential and ability to exploit elevated soil nutrient levels. Our experiment compared soil, plant and insect responses between paired carrion and control sites with spatial replication. The results of our analysis therefore provide new insight into the magnitude of the spatial contrast between carrion and its immediate surroundings. This allowed us to examine how the spatial contrast changed over time, and how carrion contributes to both spatial and temporal variation in biodiversity and ecological processes within a landscape.

## Methods

### Ethics Statement

Insects were sampled under an ACT government ‘Licence to take’ (LT2010417).

### Study Area and Experimental Design

In November 2010 (southern hemisphere spring), we added carcasses of kangaroos to 18 sites in temperate *Eucalyptus* woodland near Canberra, Australia (149°10′ East, 35°10′ South). This study area is part of the Mulligans Flat-Goorooyarroo Woodland Experiment, a long-term ecological restoration project [29]. Specific details on temperature, rainfall, floristic composition and soil types from this study area have been described by McIntyre et al [30]. We sourced carcasses of eighteen eastern grey kangaroos (*Macropus giganteus*) over a 10 day period from road kill across the city of Canberra, and positioned them at sites within 12 hours of death. Note that no animals were killed for the purposes of this study. Carcasses were only used when there was not significant blood loss. Average mass of the kangaroos was 31 kg (range 17–80 kg). Each carcass was paired with a control site 10 metres away to allow spatial comparisons between carcass and control treatments through time. We positioned each carcass (and control site) at the base a young *Eucalyptus blakelyi* tree (<2 m tall at beginning of experiment). Our study area has a history of livestock grazing, resulting in some sites being dominated by exotic grass species [30], [31], [32]. We placed half the carcasses in native-dominated grassland (*Themeda* sp. and *Austrodanthonia* sp.) and half in exotic-dominated grassland (*Phalaris* sp. and *Holcus* sp.).

### Soil and Plant Data Collection

We took a 4 cm deep soil sample (approximately 4 cm diameter) using a trowel at each carcass and control site at one week (week 0) before carcass addition, and then 12 and 26 weeks after carcass addition. Samples were taken from near the centre of each carcass. Total N content of soil and leaf samples (see below) was quantified with high temperature combustion using a LECA2000 CNS analyser [33]. We performed two analyses of total N, and used the average in our calculations. The potential mineralisable soil nitrogen (organic nitrogen) was quantified using the Illinois soil nitrogen test for amino sugar [34].

We took samples of between five and ten leaves from the tips of branches (i.e. active growth zones) in each *E. blakelyi* tree at carcass and control sites at weeks 12 and 26. No leaves were taken at week 0. Leaf nitrogen content was determined as per soil nitrogen (see above). A higher nitrogen content in leaves indicates potentially greater photosynthetic capacity, and therefore growth potential [35]. Each carcass was positioned on top of existing grasses and forbs, but these were killed off during the early stages of decay. We examined plant re-colonization of carcass patches after 52 weeks by recording the presence of grass and forb species inside a 40×40 cm quadrat at (i) the centre and (ii) edge of each carcass patch, and at (iii) the paired control site. The edge of each carcass was able to be delineated by estimation of the size of the original carcass, and the presence of a ‘halo’ of plant growth. In addition to these standardised quadrats, we also recorded the presence of all other plant species present in the remainder of a 40 cm deep perimeter around each carcass (the ‘surrounding’ vegetation) and the entire carcass patch to compile a complete inventory of grass and forb species. We collected data on plant life forms and specific leaf area (SLA) from the literature [36], [37], [38], and these are summarised in Appendix S2. SLA is the one-sided area of a fresh leaf divided by its dry mass (mm^2^.mg^−1^), and is indicative of potential relative growth rate [36]. SLA has also been shown not to vary significantly within plant species [37], thus justifying our use of data from the literature. We used the mean SLA value of each plant species presented in each published account, and excluded rare species for which data were unavailable.

### Insect Data Collection

We sampled insects using a pair of pitfall traps at each carcass and control site. We chose ants and beetles because they are an abundant and diverse components of carrion arthropod communities [3], [16], [39], and yet differ strongly in their dispersal ability and resource use. We recognise that flies are an important part of carrion arthropod communities [16], but we wanted to focus on two diverse groups with contrasting dispersal ability. Many beetles are flight-capable and have high resource specialisation [40], [41], with some specialist species requiring carrion to lay eggs and complete their life cycle. In contrast, ants are generally restricted to the ground (after post-mating dispersal), and may opportunistically scavenge from carcasses or predate on the other insects present. For these reasons, we expected beetles and ants to show very different responses to carrion. Pitfall traps were 9 cm in diameter, and were half filled with glycol solution. The traps were opened for seven days during weeks 1, 6, 12 and 26 of the experiment, which represented distinct stages of decomposition of the carcasses (see Appendix S1). We pooled pitfall trap pairs, giving n = 36 at each sampling time. All insects were extracted from the pitfall traps, stored in 70% ethanol and sorted to order and family. We further sorted beetles and ants to genus and morphospecies using relevant keys [42], [43].

### Insect Morphological Measurements

We measured body length and wing dimensions of each beetle species for comparison of mean trait values of species present at each carcass at control site. We used body length to estimate the body mass of each species of beetle using family-specific length versus weight power relationships for species of Scarabaeidae, Staphylinidae and Carabidae, and a general Coleoptera relationship for all remaining species [44]. We calculated wing loading [45] by dividing body mass by wing area (length x width), expressed as (mg/mm^2^). We calculated wing aspect ratio by dividing wing length by width, with higher ratios indicating longer and narrower wings [45]. Ant body size is correlated with microhabitat use and resource discovery rates [46], and we regarded this as a useful measure of ant dispersal ability. We measured ant head width and rear femur length, and multiplied these together to calculate a body size index for each species [47]. All morphological measurements were made on up to three individuals of each species (then averaged) using an ocular micrometer with magnification between 10× and 100×, and resolution between 0.1 and 0.01 mm, depending on the measurement and size of the insect. This approach was taken as we were interested in variation in traits between species rather than within species [48].

### Data Analysis

We were first interested in quantifying the magnitude of the contrast between carcass and control sites. We used repeated measures linear mixed models in GenStat 14 [49] to test for interactive effects of carcass addition and sampling time on total insect abundance, species richness and evenness, as well as soil and plant nitrogen content. Carcass addition and time were treated as fixed factors, and site as a random factor. We used ANOVA, also in GenStat 14, to test for differences in insect and plant species richness and evenness or traits between carcass and control treatments at a particular sampling time, with blocking by carcass-control pair. Plant SLA was log_10_ transformed, and insect abundance data were log_10_(*x*+1) transformed to meet assumptions of normality and homogeneity of variances. Percentage similarity in plant species composition between the edge and centre of carcasses was expressed as the number of shared species divided by the total number (i.e. Jaccard similarity index). We compared similarity between the centre and edge of carcasses because plant recolonisation was more likely to occur from the edge rather than from more distant control sites. We tested for effects of quadrat position (carcass centre, edge or control site) and grassland type (native or exotic) on average SLA across plant species. For this analysis, we excluded rare species that occurred only in one quadrat. We wanted to test whether insect assemblages were more homogenous in composition at carcasses than at control sites due to the widespread dominance of carrion specialists. We used Permutational Analysis of Multivariate Dispersion [50] to test for differences in among-sample heterogeneity of insect species composition between carcass and control treatments, with statistical significance determined from 10 000 permutations of the data. This test calculates the average distance to the centroid of a group of samples projected in multivariate space, with a greater distance to centroid indicating greater among-sample heterogeneity [51]. Multivariate insect abundance data were square-root transformed prior to analysis to reduce the influence of highly abundant species on sample dissimilarity. Shannon evenness was defined as E = H′/lnS, where S is the number of species in a sample, and H′ = −∑ *p_i_* ln *p_i_*, where *p_i_* is the proportion of a species in a sample which is multiplied by the natural logarithm of itself [52].

## Results

### Soil and Leaf Nitrogen

**Figure 1:**
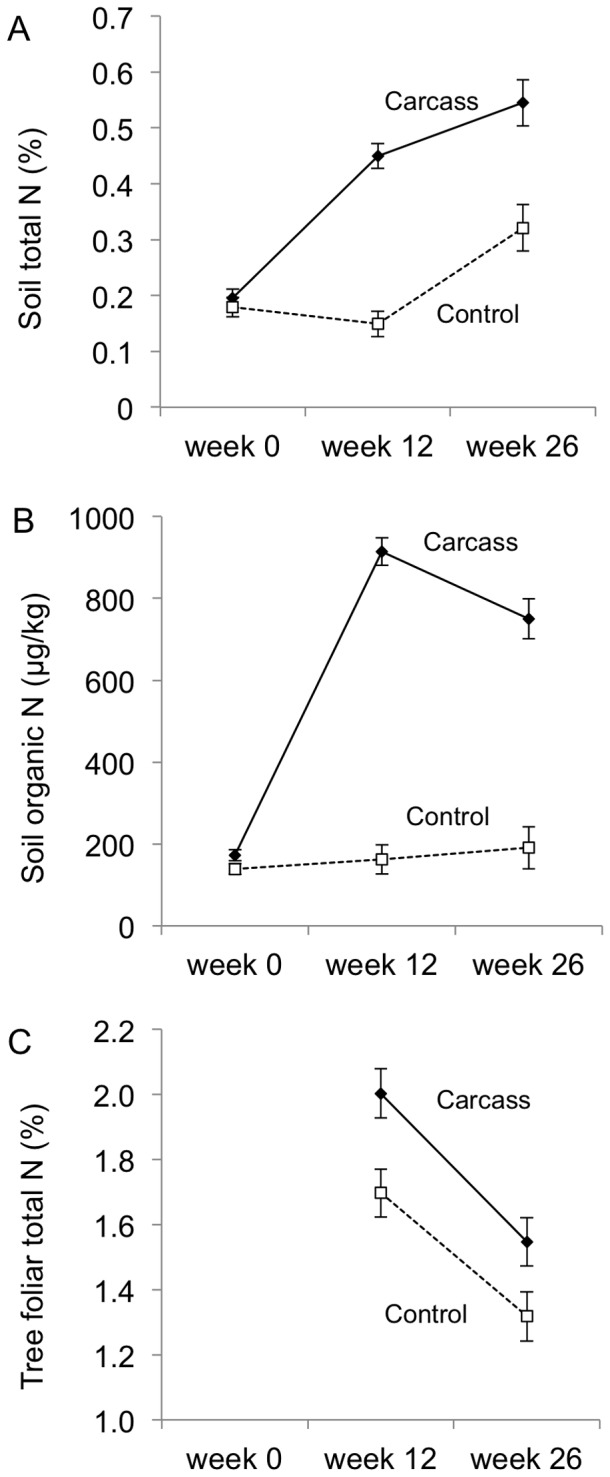
Effect of carrion on soil and foliar nitrogen. Carrion increased (a) soil total nitrogen, (b) soil organic nitrogen, and (c) tree foliar total nitrogen relative to control sites. Error bars show mean ± SE.

The addition of carrion had significant effects on soil nitrogen (Table 1). The soils in our study environment were originally low in phosphorus and nitrogen compared with grasslands improved for grazing [31], and thus have low productivity potential. The highly concentrated nutrients in each carcass (a 30 kg vertebrate carcass contains approximately one kilogram of nitrogen [5]) produced a dynamic change in soil nitrogen. We found that total soil nitrogen beneath carcasses was 200% higher than control sites after 12 weeks, and 70% higher than control sites after 26 weeks of carrion being added (Fig. 1A). In addition, the concentration of the organic fraction of soil nitrogen was 460% higher than control sites after 12 weeks, and 290% higher after 26 weeks (Fig. 1B). This nutrient pulse also was detected in plant leaf nitrogen levels from nearby tree saplings, which were 18% higher in plants near to carcasses than control sites after 12 weeks (Fig. 1C). This demonstrates the cycling of nitrogen from carcasses into soil, and the uptake and assimilation of soil nitrogen into leaf tissue during the first few weeks of decomposition.

**Table 1:** Effects of carcass treatment, time, and their interaction on soil and tree leaf nitrogen levels.

	Effects	*F*	d.f.	*P*
Soil total nitrogen (%)	Time	55.85	2	<0.001
	Treatment	29.36	1	<0.001
	Time×Treatment	37.59	2	<0.001
Soil organic nitrogen (µg/kg)	Time	136.31	2	<0.001
	Treatment	26.68	1	<0.001
	Time×Treatment	107.28	2	<0.001
Tree foliar nitrogen (%)	Time	29.73	1	<0.001
	Treatment	12.71	1	<0.001
	Time×Treatment	0.26	1	0.609

### Plant Assemblage Richness and Composition

**Figure 2:**
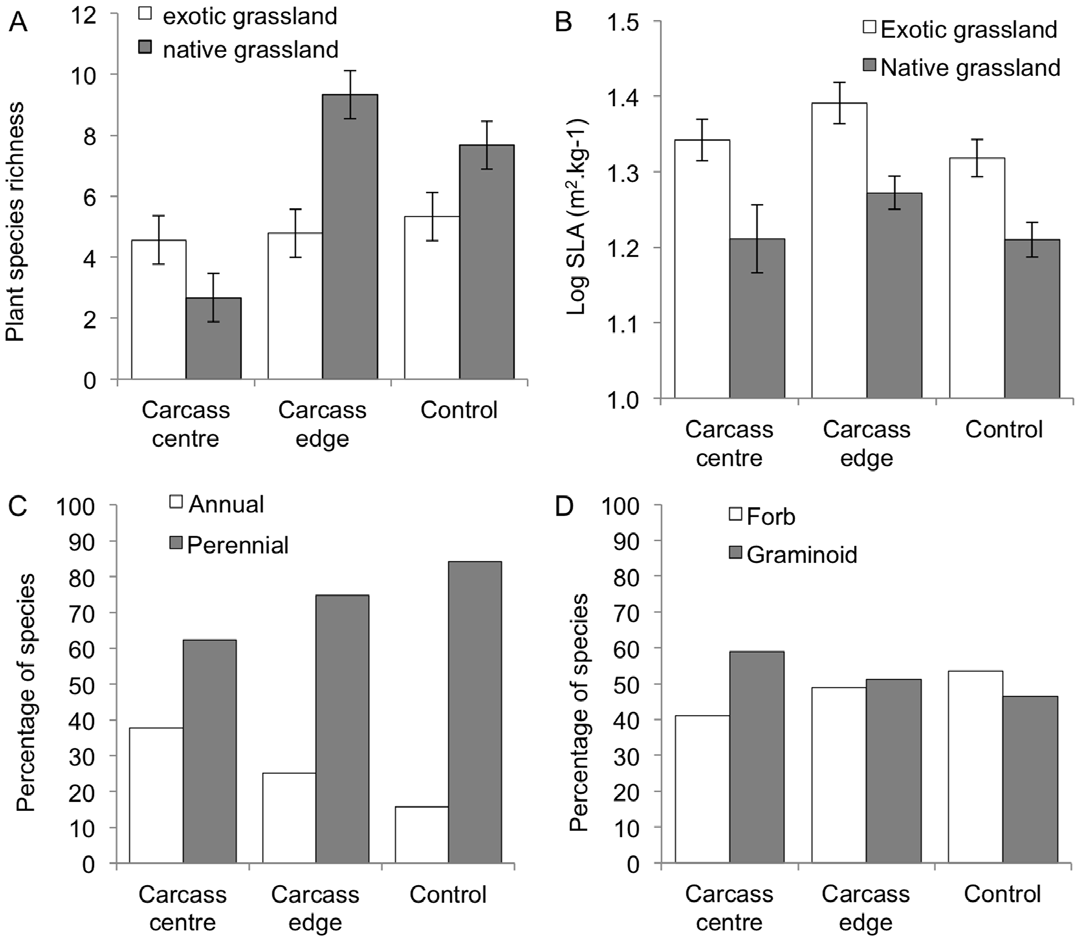
Differences in plant species richness and traits between carcass centre, carcass edge, and control sites. (a) Species richness of plants species in exotic grassland compared with native grassland. (b) Specific leaf area of plants in exotic grassland compared with native grassland. (c) Percentage of annuals versus perennials. (d) Percentage of graminoids versus forbs. Error bars show mean ± SE.

**Figure 3:**
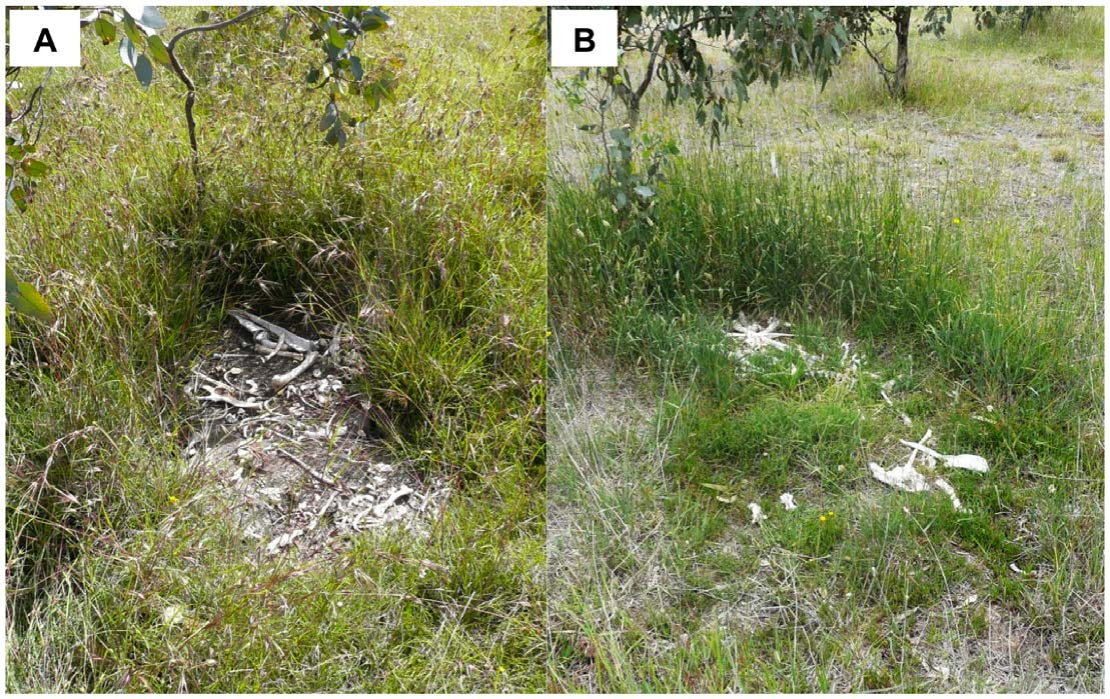
Recolonisation of plants 52 weeks after carrion addition. Very little recolonisation had occurred in the native-dominated grassland sites (a), whereas re-colonisation had progressed further in all exotic-dominated grassland sites (b), reflecting greater tolerance of some exotic species to extremely high nutrients. These examples indicate a different succession trajectory for native versus exotic grassland plant assemblages, with recolonisation occurring over different time scales.

Plant recolonisation of carcasses was faster in the exotic-dominated grassland where, after 52 weeks, the composition of plants in the centre of each carcass patch more closely matched the composition of species at the edge of each carcass patch in the exotic-dominated grassland (56% shared species) than in native-dominated grassland (37% shared species). We found a significant interaction between carcass treatment and grassland type (*F*_2_ = 11.48, *P*<0.001) and their effect on plant species richness at carcass patches after 52 weeks. Plant species richness was similar across carcass and control sites in the exotic grassland, but the number of species in the centre of carcass patches in native grassland was only a third that of species at the edge of carcass patches or at control sites (Fig. 2A). This result is further highlighted by our observation that four of the nine native-dominated carcass patches had no re-colonising plants at all, whereas all nine of the exotic-dominated carcass patches had re-colonising plants 52 week after carrion was added (compare Fig. 3A with 3B). The rapid response of plants in the exotic grassland was associated with a significantly higher specific leaf area among the constituent plant species (Fig. 2B, *F*_1_ = 18.03, *P*<0.001). Regardless of grassland type, plants recolonising the centre of carcass patches tended to be annuals rather perennials, and graminoids rather than forbs (Fig. 2C, 2D).

### Insect Assemblage Diversity and Composition

**Figure 4:**
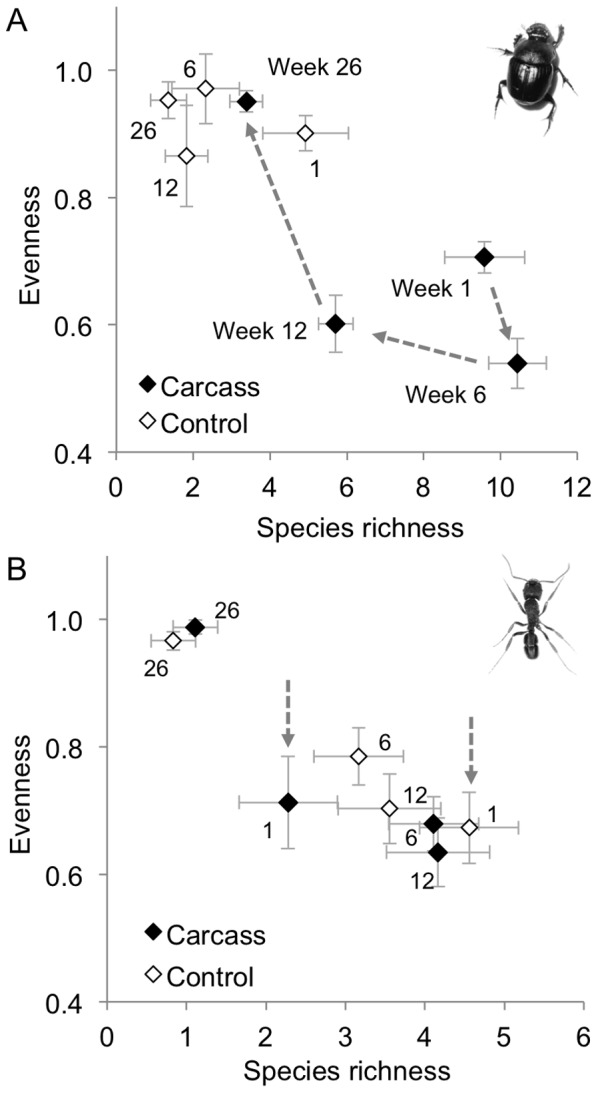
Effect of carrion on beetle and ant diversity. (a) Species richness and evenness of beetles at carcasses showed a trajectory through time clearly different from control sites at weeks 1, 6 and 12, with a return to similar levels at week 26 (blue arrows). (b) Species richness of ants at carcasses was half that at control sites during week 1 (red arrows), but was then similar to control sites at week 6, 12 and 26 of the experiment. Error bars show mean ± SE.

**Figure 5:**
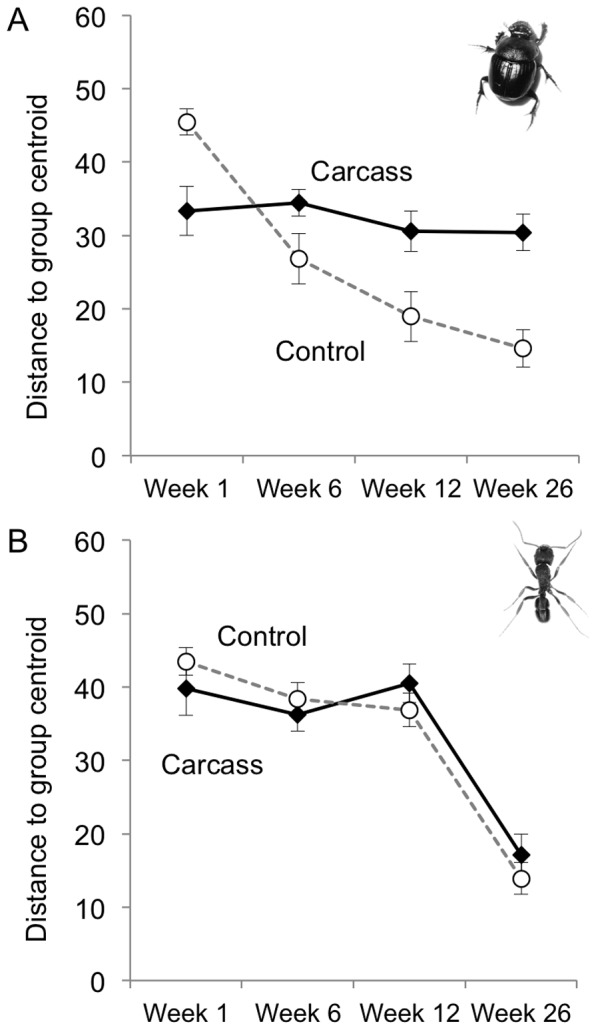
Effect of carrion on insect assemblage heterogeneity. Average distance to group centroid, reflecting heterogeneity in composition among samples of (a) beetles and (b) ants from carrion and control treatments. Error bars show mean ± SE.

We sampled a total of 33 ant species (3330 individuals) and 119 beetle species (3614 individuals) from all carcass and control sites over the four sampling times (Appendix S3 and S4). Beetle and ant assemblages displayed contrasting dynamic responses to carrion. We found that beetle assemblages had a significantly higher species richness and lower evenness during early stages of decay, with a gradual return to levels similar to control sites by week 26 (Fig. 4A, Table 2). This contrasted strongly with ant assemblages, which initially had lower species richness at carcass sites, and did not show the same decrease in evenness as the beetle assemblage (Fig. 4B). We found an overall significant difference in among-sample heterogeneity from carcass and control sites at each sample times for both beetles (*F* = 21.11, *P*<0.001) and ants (*F* = 11.81, *P*<0.001). However, comparisons between carcass and control sites for each sampling time showed distinctive and contrasting patterns for the beetles and ants (Fig. 5). In the early weeks of decomposition, the composition of beetle assemblages at carrion sites was less variable than at control sites, indicating spatial monopolisation of carcass patches by a few abundant species (Fig. 5A). This changed as decay progressed, with the beetle assemblage becoming more heterogeneous at carrion sites relative to control sites. This was due to widespread species at carcasses becoming less dominant, while assemblages at control sites showed a seasonal decrease in heterogeneity (Fig. 5A). In contrast, there was never a difference in ant assemblage heterogeneity between carcass and control sites (Fig. 5B), with the overall significant difference among groups due a decrease in heterogeneity in week 26 in late autumn. This indicated there was no spatial monopolisation of carcasses by a few ant species, in contrast to that which we observed for beetles.

**Table 2:** Effects of carcass treatment, time, and their interaction on (A) ant and (B) beetle abundance, species richness and evenness.

		Effects	F	d.f.	P
(A)	Abundance	Time	48.81	3	<0.001
		Treatment	3.25	1	0.080
		Time×Treatment	4.55	3	0.009
	Species richness	Time	26.05	3	<0.001
		Treatment	3.35	1	0.076
		Time×Treatment	4.51	3	0.009
	Evenness	Time	44.38	3	<0.001
		Treatment	1.08	1	0.322
		Time×Treatment	1.41	3	0.264
(B)	Abundance	Time	40.09	3	<0.001
		Treatment	230.92	1	<0.001
		Time×Treatment	19.05	3	<0.001
	Species richness	Time	21.46	3	<0.001
		Treatment	70.07	1	<0.001
		Time×Treatment	11.51	3	<0.001
	Evenness	Time	50.49	3	<0.001
		Treatment	28.86	1	<0.001
		Time×Treatment	12.83	3	<0.001

### Insect Dispersal Traits

**Figure 6:**
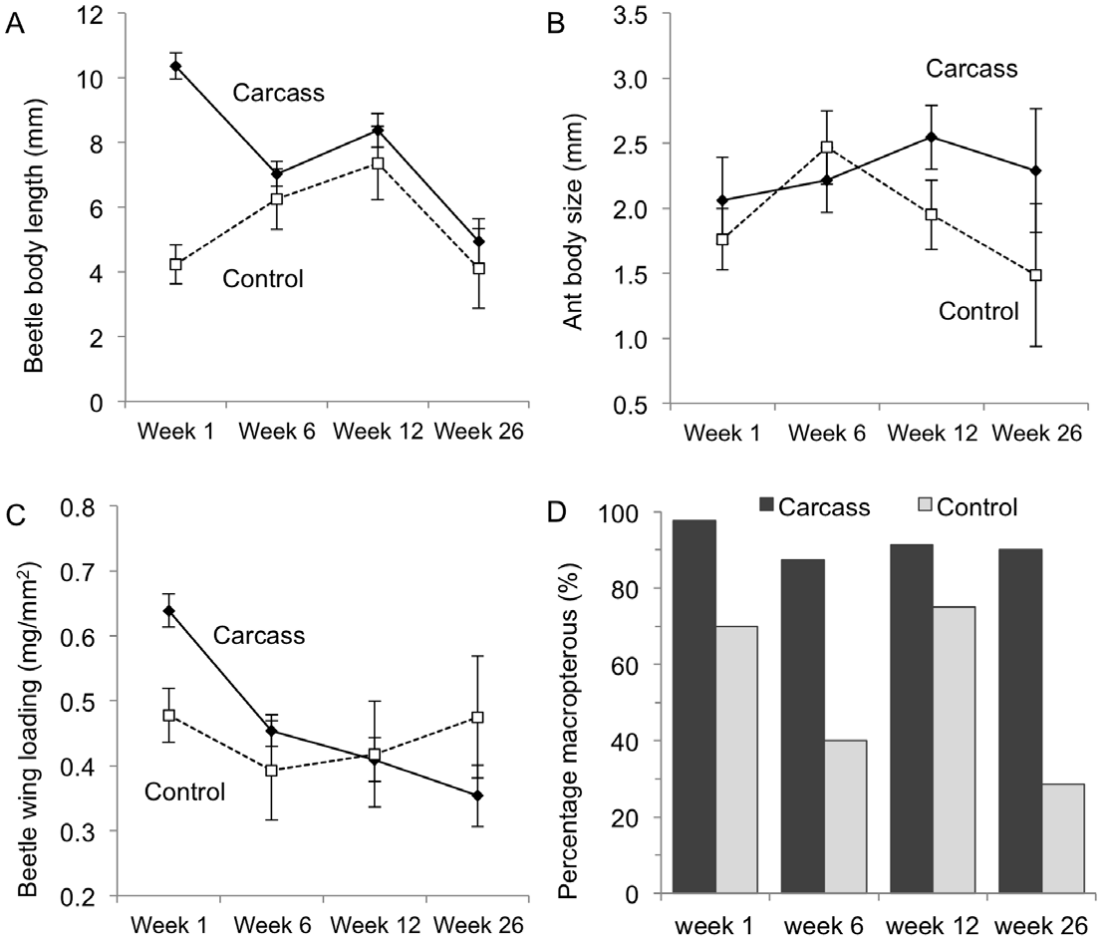
Differences in insect morphological traits between carcass and control sites. (a) Beetle species at carrion during week 1 were larger on average than those at control sites. (b) Beetle species at carrion during week 1 had higher wing loadings on average than beetle species at control sites. (c) No differences in ant body size were observed at different stages of carrion decomposition. (d) Macropterous beetles were always more prevalent at carcass sites that control sites. Error bars show mean ± SE.

The contrasting response of beetle and ant assemblages was associated with their different dispersal abilities, with significant differences in morphological traits across beetle species but not ant species (Table 3). Beetle species at carrion were larger than species at control sites one week after carrion was added (Fig. 6A), whereas ants showed no significant difference in body size at any of the four sampling times (Fig. 6B). Beetles at carcasses also had significantly higher wing loading, but only at one week after carrion was added (Fig. 6C). We also found that a higher percentage of beetles at carcasses had functional wings compared with beetles at control sites (Fig. 6D).

**Table 3:** Effects of carcass treatment, time, and their interaction on insect morphological traits.

	Effect	*F*	d.f.	*P*
(A) Ant body size	Time	0.99	3	0.395
	Treatment	0.09	1	0.769
	Time×Treatment	0.62	3	0.600
(B) Beetle body length	Time	12.42	3	<0.001
	Treatment	39.37	1	<0.001
	Time×Treatment	8.82	3	<0.001
(C) Beetle wing loading	Time	9.25	3	<0.001
	Treatment	1.81	1	0.179
	Time×Treatment	1.64	3	0.180

## Discussion

Our results show that carrion in ecosystems represents a dynamic driver of species richness and nutrient cycling. This is supported by our observations, which showed elevated nitrogen in soil and plant leaf tissue at carrion patches, different rates of recolonization by plants, and a diverse and functionally distinct set of insects occurring at carcasses compared with control sites. Importantly, we have shown that the pattern of response by insects and plants was associated with particular functional traits that allow them to respond to and then exploit carrion. This builds on previous work that has demonstrated separately the effects of carrion on soil nutrient heterogeneity [53], scavenger community structure [16], [54], and plant composition [18], and develops further our understanding of how carrion generates spatial and temporal variation in ecological communities.

We found that the nitrogen spike in soils translated into an increase in leaf nitrogen in the nearby *E. blakelyi* trees, indicating the movement of nutrients from the decomposing carcasses into live plant tissue via the soil subsystem [15], [25]. Trees in the genus *Eucalyptus* are not nitrogen-fixing and must use available nitrogen in the soil. This suggests that the increase in soil nitrogen due to carcass decomposition was actively taken up by the trees we sampled. The differences we detected in foliage nitrogen between carcass and control sites was smaller than that reported from analogous experimental studies in North America [15], [28], but this may be due to the very different plant species involved, as well as underlying microbial and soil properties. The dramatic change in soil nitrogen also resulted in a greater dominance of grass species with high average specific leaf area. It is well established that these functional plant types typically respond to elevated nitrogen [31]. This reflects observations in a tallgrass prairie ecosystem of North America, where forbs decreased in cover at the centre of ungulate carcasses after 1 year, but grasses increased in cover [18]. This is evidence of *in situ* organisms displaying trait-dependent responses to new nutrient-rich resource patches. The effects of carcasses on the diversity and composition of ground-layer plant assemblages depended on whether the carcasses were placed in native- or exotic-dominated grassland. Plants at the carcass treatment sites were initially killed by the carcasses due to prolonged shading by carcass remains and rapid soil chemistry changes, but later recolonised from the edges. The quick recolonization of carrion patches by plants in the exotic grassland was largely due to seeding, except for one species that used stolons (*Cynodon dactylon*). This underlines a context-dependent effect of carrion not previously described at local scales. This parallels a pattern observed for the effects of migrating salmon (*Oncorhynchus* spp.) carcasses on plants in North America, with effects reduced in the more highly productive catchments [27]. Together the results suggest a general principle of context-dependency resulting in temporally attenuated effects of carrion in high productivity ecosystems or in habitats with a high proportion of nutrient-responsive plant species.

We showed that dispersal traits of insects were associated with their responses to ephemeral resource opportunities. The distinct dispersal traits of beetles compared with ants was likely to explain, in part, their contrasting dynamics in assemblage composition, and led to their changes in spatial heterogeneity during different stages of carcass decomposition. Dispersal plays a key role in insect spatial diversity patterns [55], and our study provides a further example relevant to carrion. Although carrion affected the relative abundance of ants present in the landscape, it did not affect species richness or spatial heterogeneity. The lack of flight to facilitate rapid foraging means that ants are generally less able to move as rapidly as flight-capable beetles, regardless of any resource specialisation they might have. Although we found that some ant species dominated individual carcasses, such as the meat ant *Iridomyrmex purpureus*, there were no species that monopolised all the carcasses in the same way some beetle species did. This result is due, in part, to the lack of strong specialisation of ants on carrion resources, with scavenging more likely to be opportunistic. For beetles, capacity for flight and higher wing-aspect ratios were associated with beetles at carrion generally. A larger body size and higher wing loading were important functional traits that were associated with the early colonisation of fresh carrion and spatial monopolisation of this resource. Beetles visiting fresh carcasses were typically equipped with wings that enable manoeuvrable flight [45], suggesting that they can actively seek carrion, and with their large body size have the metabolic capacity to do so over greater distances than smaller beetles. These traits are exemplified by the carrion specialists *Ptomaphila lacrysoma* and *Creophilus erythrocephalus*, which are well known to frequent carrion in south-eastern Australia [56], [57]. These species, as well as several others, were not recorded at control sites (Appendix S4), and have not been recorded as part of previous research in the study area [48]. This highlights the unique composition of carrion insect assemblages. Ants at carrion displayed no characteristic morphologies during different stages of carrion decay, and this matched their lack of difference in spatial heterogeneity between carcasses or control sites. Although we focused on morphological traits related to dispersal ability, it is important to point out that the feeding traits of insects are equally important in determining their responses to carrion [16]. For example, the carrion specialists mentioned above also feed on the flesh of carcasses or prey on the insects that do. Their early arrival at a carcass is therefore critical if they are to exploit a resource that will quickly be exhausted. Further research is needed to test if dispersal limitation or small body size actually prevents some species from exploiting carrion, as this would provide a mechanistic link between morphology and assemblage responses to carrion resources.

Together, our findings provide an integrated example of how traits of opportunist species in two different taxonomic groups are associated with their ability to exploit patchy and dynamic resources. This adds to the literature on carrion resource specialisation traits and temporal assemblage dynamics [16], [23], to give a fuller picture of the processes that drive spatial changes in species composition. It also supports previous claims that carrion might need to be managed in some ecosystems to maintenance of biodiversity and ecological processes [9], [58]. This includes, for example, the role of carrion in the persistence of dependent insects [59] or the nutrient opportunities it can provide to plants [15]. Our study indicates that appropriate consideration of spatial and temporal continuity in carrion will enable insects of variable dispersal ability to exploit this resource, and will promote spatial heterogeneity in nutrient cycling and plant communities.

## Supporting Information

Appendix S1 Stages of decomposition when insects were sampled.
(DOCX)

Appendix S2 Summary of plant species and their traits.
(DOCX)

Appendix S3 Summary of ant species.
(DOCX)

Appendix S4 Summary of beetle species.
(DOCX)
