# Supplementary material for: Species Traits Predict Assemblage Dynamics at Ephemeral Resource Patches Created by Carrion
Source: PLoS One. 2013 Jan 11;8(1):e53961. doi: 10.1371/journal.pone.0053961 (PMC3543354; doi:10.1371/journal.pone.0053961)
Supplement: Appendix S1 — Stages of decomposition when insects were sampled. (DOCX) [file pone.0053961.s001.docx]

**Appendix S1**. Insects were sampled at four intervals during the experiment. (a) week 1 – bloat to active decay, (b) week 6 - advanced decay, (c) week 12 - dry decay, (d) week 26 – remains only. Loss of biomass was most rapid during the first two weeks when fluids and soft tissue was lost to the soil and consumed by fly larvae. Recalcitrant parts of the carcasses (skin, fur, stomach contents) remained for many weeks after.
