# Supplementary material for: Species Traits Predict Assemblage Dynamics at Ephemeral Resource Patches Created by Carrion
Source: PLoS One. 2013 Jan 11;8(1):e53961. doi: 10.1371/journal.pone.0053961 (PMC3543354; doi:10.1371/journal.pone.0053961)
Supplement: Appendix S2 — Summary of plant species and their traits. (DOCX) [file pone.0053961.s002.docx]

**Appendix S2**. Summary of plant species and their traits sampled at carcass and control sites one year after the addition of carcasses. Data on plant life forms and specific leaf area were collated from the literature (see main article for references).

| **Plant species** | **Mean SLA (common species only)** | **Exotic or Native** | **Annual or Perennial** | **Graminoid or forb** |
| --- | --- | --- | --- | --- |
| *Acaena ovina* | 16.60 | n | p | f |
| *Acetosella vulgaris* | 20.57 | e | p | f |
| *Aira spp.* | 27.80 | e | a | g |
| *Alternanthera sp.* |  | n | p | f |
| *Aristida ramosa* | 8.21 | n | p | g |
| *Arthropodium sp.* |  | n | p | f |
| *Austrodanthonia spp.* | 15.52 | n | p | g |
| *Austrostipa bigeniculata* |  | n | p | g |
| *Bothriochloa macra* | 22.70 | n | p | g |
| *Briza minor* | 37.74 | e | a | g |
| *Bromus hordeaceus* | 25.86 | e | a | g |
| *Carex inversa* | 16.22 | n | p | g |
| *Centaurium erythraea* | 27.22 | e | p | f |
| *Centaurium tenuiflorum* |  | e | a | f |
| *Chamaesyce drummondii* |  | n | p | f |
| *Chrysocephalum apiculatum* |  | n | p | f |
| *Cirsium vulgare* | 19.05 | e | a | f |
| *Conyza sp.* |  | e | a | f |
| *Cymbonotus lawsonianus* |  | n | p | f |
| *Cynodon dactylon* | 29.71 | n | p | g |
| *Daucus glochidiatus* | 15.14 | n | a | f |
| *Dichelachne rara* |  | n | p | g |
| *Elymus scaber* | 19.95 | n | p | g |
| *Eragrostis brownii* |  | n | p | g |
| *Euchiton gymnocephalus* | 21.86 | n | p | f |
| *Euchiton sphaericus* |  | n | a | f |
| *Gamochaeta americana* |  | e | a | f |
| *Gonocarpus tetragynus* | 8.36 | n | p | f |
| *Goodenia hederacea* |  | n | p | f |
| *Haloragis heterophylla* |  | n | p | f |
| *Holcus lanatus* | 30.00 | e | p | g |
| *Hydrocotyle sp.* |  | n | p | f |
| *Hypericum gramineum* | 15.00 | n | p | f |
| *Hypochaeris glabra* | 12.37 | e | a | f |
| *Hypochaeris radicata* | 23.43 | e | p | f |
| *Joycia pallida* | 6.56 | n | p | g |
| *Juncus sp.* | 4.58 | n | p | g |
| *Lachnogrostis filiformis* |  | n | a | g |
| *Leptorhynchos squamatus* |  | n | p | f |
| *Linum trigynum* |  | e | a | f |
| *Lolium spp.* | 19.23 | e | a | g |
| *Microlaena stipoides* | 19.52 | n | p | g |
| *Moenchia erecta* |  | e | a | f |
| *Oxalis perennans* | 57.54 | n | p | f |
| *Panicum effusum* | 12.59 | n | p | g |
| *Persecaria prostrata* |  | n | p | f |
| *Phalaris aquatica* | 23.40 | e | p | g |
| *Plantago lanceolata* | 19.50 | e | p | f |
| *Rumex brownii* | 30.86 | n | p | f |
| *Schoenus apogon* | 9.96 | n | p | g |
| *Senecio quadridentatus* | 15.49 | n | p | f |
| *Solenogyne dominii* | 17.46 | n | p | f |
| *Themeda triandra* | 19.41 | n | p | g |
| *Tolpis umbellata* |  | e | a | f |
| *Tricoryne elatior* | 21.80 | n | p | f |
| *Trifolium spp.* | 24.68 | e | a | f |
| *Vittadinia muelleri* |  | n | p | f |
| *Vulpia spp.* | 20.76 | e | a | g |
| *Wahlenbergia spp.* |  | n | p | f |
