# Supplementary material for: Species Traits Predict Assemblage Dynamics at Ephemeral Resource Patches Created by Carrion
Source: PLoS One. 2013 Jan 11;8(1):e53961. doi: 10.1371/journal.pone.0053961 (PMC3543354; doi:10.1371/journal.pone.0053961)
Supplement: Appendix S3 — Summary of ant species. (DOCX) [file pone.0053961.s003.docx]

**Appendix S3**. Summary of ant species sampled at carcass and control sites at four different times. Counts at control sites are shown in brackets.

| Subfamily | Genus/Species | Week 1 | Week 6 | Week 12 | Week 26 |
| --- | --- | --- | --- | --- | --- |
| Dolichoderinae | *Iridomyrmex purpureus* | 153 (37) | 34 (17) | 28 (17) | 1 |
| Dolichoderinae | *Iridomyrmex sp1* | 200 (393) | 345 (134) | 418 (199) | 4 (2) |
| Dolichoderinae | *Iridomyrmex sp2* | 18 (2) | 7 (3) | 1 (7) | 1 (1) |
| Dolichoderinae | *Iridomyrmex sp3* | 5 |  |  |  |
| Dolichoderinae | *Iridomyrmex sp4* | (47) | 3 (8) | 3 |  |
| Dolichoderinae | *Iridomyrmex sp5* |  | (4) | (2) |  |
| Dolichoderinae | *Leptomyrmex sp1* |  |  |  | 2 |
| Formicinae | *Camponotus consobrinus* | 3 | 12 (23) | 27 (5) | 2 (1) |
| Formicinae | *Camponotus sp1* | 3 (25) | 5 | 2 |  |
| Formicinae | *Camponotus sp2* | 4 (10) | 4 (1) | 7 (2) | 1 |
| Formicinae | *Melophorus sp1* | (24) | 32 (8) | 24 (10) |  |
| Formicinae | *Melophorus sp2* | (1) | 4 |  |  |
| Formicinae | *Melophorus sp3* |  |  | 1 |  |
| Formicinae | *Notoncus sp1* | (33) | 4 (6) | 34 (24) | 3 (3) |
| Formicinae | *Paratrechina sp1* | 1 (6) |  | (2) | 1 |
| Formicinae | *Paratrechina sp2* | 10 (64) | 23 (4) | 9 (2) | 1 (2) |
| Formicinae | *Paratrechina sp2* |  |  | (1) |  |
| Myrmeciinae | *Myrmecia sp1* |  |  | 1 (1) |  |
| Myrmicinae | *Meranoplus sp1* | (2) |  |  |  |
| Myrmicinae | *Monomorium sp1* | 3 |  |  |  |
| Myrmicinae | *Monomorium sp1* |  |  | 1 (15) | 1 |
| Myrmicinae | *Monomorium sp2* | 14 (155) | 59 (38) | 116 (24) | (2) |
| Myrmicinae | *Phiedole sp1* | 31 (34) | 17 (28) | 21 (58) | 3 (9) |
| Myrmicinae | *Phiedole sp2* | (1) |  |  |  |
| Myrmicinae | *Phiedole sp3* | 1 | (8) | 1 (3) |  |
| Myrmicinae | *Phiedole sp4* | (1) | 7 (4) | 7 (19) | 5 |
| Myrmicinae | *Phiedole sp5* |  | 15 | 4 (1) | 1 |
| Myrmicinae | *Solenopsis sp1* | 3 (2) | (1) | 5 |  |
| Myrmicinae | *Tetramorium sp1* | (1) |  | 1 |  |
| Ponerinae | *Cerapachys sp1* | 1 (1) |  |  |  |
| Ponerinae | *Heteroponera sp1* | (2) | 1 (1) | (1) |  |
| Ponerinae | *Heteroponera sp2* | (1) |  |  |  |
| Ponerinae | *Rhytidoponera metallica* | (5) | 6 | 5 (3) |  |
