# Supplementary material for: Species Traits Predict Assemblage Dynamics at Ephemeral Resource Patches Created by Carrion
Source: PLoS One. 2013 Jan 11;8(1):e53961. doi: 10.1371/journal.pone.0053961 (PMC3543354; doi:10.1371/journal.pone.0053961)
Supplement: Appendix S4 — Summary of beetle species. (DOCX) [file pone.0053961.s004.docx]

**Appendix S4**. Summary of beetle species sampled at carcass and control sites at four different times. Counts at control sites are shown in brackets.

| Family | Genus/Species | Week 1 | Week 6 | Week 12 | Week 26 |
| --- | --- | --- | --- | --- | --- |
| Anobiidae | *Ptinus sp1* | (1) | (1) |  |  |
| Anthicidae | *Formicomus sp3* | (2) | 1 |  |  |
| Anthicidae | *Formicomus sp2* | 15 (6) | 104 (4) | 14 (1) | 3 (1) |
| Anthicidae | *Formicomus sp1* | 2 (19) | 2 (2) | 7 (1) | 2 |
| Bostrichidae | unknown sp |  |  | 1 |  |
| Byrrhidae | *Microchaetes sp1* | (2) | (1) | 4 (2) | (1) |
| Carabidae | unknown sp |  | 1 | 1 |  |
| Carabidae | *Scopodes sp1* | (2) |  |  |  |
| Carabidae | *Prosopogmus sp1* |  |  | 2 |  |
| Carabidae | *Promecoderus sp1* |  |  |  | (1) |
| Carabidae | *Notagonum sp2* | 1 | 1 |  | 1 |
| Carabidae | *Notagonum sp1* |  | 1 | 5 (3) | 3 |
| Carabidae | *Neocarenum sp2* | (1) | 7 |  |  |
| Carabidae | *Neocarenum sp1* | (1) |  |  |  |
| Carabidae | *Hypharpax sp1* | 2 | 3 (1) |  |  |
| Carabidae | *Diaphoromerus sp1* |  | (1) |  |  |
| Carabidae | *Carenum sp1* |  | 1 |  |  |
| Carabidae | *Amblystomus sp1* |  | (2) |  |  |
| Chrysomelidae | unknown sp |  |  | (1) | 2 (1) |
| Chrysomelidae | unknown sp |  | 1 |  |  |
| Chrysomelidae | unknown sp | (3) | 8 (4) | (1) |  |
| Chrysomelidae | unknown sp |  |  | (1) |  |
| Chrysomelidae | *Longitarsus sp1* | (1) | (1) |  |  |
| Cleridae | unknown sp |  | 1 |  |  |
| Cleridae | *Phlogistus sp1* | (1) |  |  |  |
| Cleridae | *Necrobia sp2* | 6 | 3 |  | 2 |
| Cleridae | *Necrobia sp1* | 1 |  |  |  |
| Coccinellidae | unknown sp |  | 1 |  |  |
| Coccinellidae | *Rhyzobius sp1* | (1) | (1) |  |  |
| Coccinellidae | *Diomus sp3* |  |  |  | 1 |
| Coccinellidae | *Diomus sp2* |  | 1 |  |  |
| Coccinellidae | *Diomus sp1* | (1) |  |  |  |
| Corylophidae | *Sericoderus sp2* | 1 | 2 |  |  |
| Corylophidae | *Sericoderus sp1* |  | (1) |  |  |
| Curculionidae | unknown sp |  |  |  | (1) |
| Curculionidae | *Naupactus leucoloma* |  |  | 1 |  |
| Curculionidae | *Listroderus sp1* |  |  |  | (1) |
| Curculionidae | *Emplesis sp1* | (1) |  |  |  |
| Curculionidae | *Cubicorhynchus sp1* |  | 1 (1) | 2 (3) |  |
| Curculionidae | *Baeosomus sp1* |  | (1) |  |  |
| Curculionidae | *Anorthorhinus sp1* |  |  | 8 |  |
| Dermestidae | *Dermestes sp1* | 30 | 8 |  |  |
| Elateridae | *Conoderus sp1* |  | 1 |  |  |
| Elateridae | *Agrypnus sp5* |  |  |  | (1) |
| Elateridae | *Agrypnus sp4* | (5) | 1 |  |  |
| Elateridae | *Agrypnus sp3* | 1 (1) |  |  |  |
| Elateridae | *Agrypnus sp2* | (1) |  |  |  |
| Elateridae | *Agrypnus sp1* | 6 (32) | 10 | 2 (2) |  |
| Histeridae | *Saprinus sp3* | 1 |  |  |  |
| Histeridae | *Saprinus sp2* | 607 (11) | 35 | 46 |  |
| Histeridae | *Saprinus sp1* | 9 |  |  |  |
| Hybosoridae | unknown sp | (4) |  |  |  |
| Hybosoridae | unknown sp | (1) |  |  |  |
| Latridiidae | unknown sp | 1 |  |  |  |
| Latridiidae | *Cortinicara sp2* |  |  |  | 3 |
| Latridiidae | *Cortinicara sp1* | (2) |  |  |  |
| Leiodidae | unknown sp | 3 |  |  | 5 |
| Melyridae | *Dicranolaius sp2* |  | 1 |  |  |
| Melyridae | *Dicranolaius sp1* |  |  | 1 |  |
| Nitidulidae | unknown sp | 1 |  |  |  |
| Phalacridae | unknown sp | 1 | 1 |  |  |
| Pselaphidae | unknown sp |  |  | 1 |  |
| Pselaphidae | *Tyraphus sp1* | (1) | 1 (1) | 1 | 3 |
| Pselaphidae | *Rybaxis sp1* | 1 (2) |  |  |  |
| Pselaphidae | *Pselaphaulax sp1* | (3) |  |  | 1 |
| Pselaphidae | *Eupines sp1* | (2) |  | 1 | (2) |
| Ptilidae | *Actinopteryx sp1* | 1 | 14 | 2 |  |
| Scarabaeidae | unknown sp |  |  | (1) |  |
| Scarabaeidae | unknown sp |  | (1) | 2 |  |
| Scarabaeidae | unknown sp |  | (1) |  |  |
| Scarabaeidae | unknown sp |  | (2) |  |  |
| Scarabaeidae | unknown sp |  | 1 (1) |  |  |
| Scarabaeidae | unknown sp | (1) | (1) |  |  |
| Scarabaeidae | unknown sp | 1 |  |  |  |
| Scarabaeidae | *Onthophagus sp5* |  | 2 |  |  |
| Scarabaeidae | *Onthophagus sp4* |  | 1 |  |  |
| Scarabaeidae | *Onthophagus sp3* | 9 | 13 | 5 |  |
| Scarabaeidae | *Onthophagus sp2* | 3 | 3 |  |  |
| Scarabaeidae | *Onthophagus sp1* | 7 | 5 |  |  |
| Scarabaeidae | *Euoniticellus sp3* |  | 8 |  |  |
| Scarabaeidae | *Automolius sp2* | 1 |  |  |  |
| Scarabaeidae | *Automolius sp1* |  |  | (2) |  |
| Scarabaeidae | *Automolius sp1* |  |  | (1) |  |
| Scarabaeidae | *Aphodius sp4* |  |  | 65 (9) |  |
| Scarabaeidae | *Aphodius sp3* | 67 | 20 |  |  |
| Scarabaeidae | *Aphodius sp2* | 6 (3) | 2 (1) | (2) | 1 |
| Scarabaeidae | *Aphodius sp1* |  |  | 1 |  |
| Scydmaenidae | *Euconnus sp3* |  |  |  | 2 |
| Scydmaenidae | *Euconnus sp2* |  |  |  | (1) |
| Scydmaenidae | *Euconnus sp1* | (4) | 3 |  |  |
| Silphidae | *Ptomaphila lacrysoma* | 84 | 16 |  |  |
| Staphylinidae | unknown sp | 1 |  |  | 1 |
| Staphylinidae | unknown sp | 3 |  |  |  |
| Staphylinidae | *Quedius sp1* | 3 | 1 |  |  |
| Staphylinidae | *Polylobus sp4* |  |  |  | 17 |
| Staphylinidae | *Polylobus sp3* | (1) |  |  |  |
| Staphylinidae | *Polylobus sp2* | 2 (1) |  |  |  |
| Staphylinidae | *Polylobus sp1* | 9 (3) | 1 | 1 | 5 (10) |
| Staphylinidae | *Philonthus sp3* | 4 | 3 |  |  |
| Staphylinidae | *Philonthus sp2* | 3 |  |  |  |
| Staphylinidae | *Philonthus sp1* | 10 (1) | 4 |  |  |
| Staphylinidae | *Paederus sp1* | 3 (8) |  |  |  |
| Staphylinidae | *Ocalea sp1* | (1) |  |  | (1) |
| Staphylinidae | *Heterothops sp1* | (1) |  |  | 1 |
| Staphylinidae | *Creophilus erythrocephalus* | 122 | 10 |  |  |
| Staphylinidae | *Atheta sp1* | (9) | 4 |  | 10 (1) |
| Staphylinidae | *Anotylus sp3* | 4 | 2 |  |  |
| Staphylinidae | *Anotylus sp2* |  | 57 |  | 8 |
| Staphylinidae | *Anotylus sp1* |  |  | 3 |  |
| Tenebrionidae | unknown sp |  | 1 |  |  |
| Tenebrionidae | unknown sp |  | 3 |  |  |
| Tenebrionidae | unknown sp | 1 | 23 (1) |  |  |
| Tenebrionidae | unknown sp | 1 | 1 |  | 1 |
| Tenebrionidae | *Helea sp1* |  | (1) |  |  |
| Tenebrionidae | *Csiro nigra* | 1 (1) | 5 (1) |  |  |
| Trogidae | *Omorgus sp3* | 1 | 1 |  |  |
| Trogidae | *Omorgus sp2* | 24 | 32 | 29 | 6 |
| Trogidae | *Omorgus sp1* | 297 (1) | 856 (2) | 445 (1) | 10 |
| Trogidae | *Omorgus scaber* |  | 1 |  |  |
